# Cross-sectional study of pediatric pain prevalence, assessment, and treatment at a Canadian tertiary hospital

**DOI:** 10.1080/24740527.2021.1961081

**Published:** 2021-09-29

**Authors:** Alex Senger, Rhonda Bryce, Casey McMahon, Krista Baerg

**Affiliations:** a College of Medicine, University of Saskatchewan, Saskatoon, Saskatchewan, Canada; b Clinical Research Support Unit, College of Medicine, University of Saskatchewan, Saskatoon, Saskatchewan, Canada; c Department of Pediatrics, College of Medicine, University of Saskatchewan, Saskatoon, Saskatchewan, Canada; d Saskatchewan Health Authority–Saskatoon, Saskatoon, Saskatchewan, Canada

**Keywords:** pediatrics, hospital medicine, pain, procedural, pain management, lidocaine, sucrose, self report, quality improvement, pain assessment

## Abstract

**Background:**

Painful experiences are common among hospitalized children. Long-term negative biopsychosocial consequences of undertreated pain are recognized.

**Aims:**

The study benchmarks pain prevalence, assessment, and treatment as first steps to improve pain care in a Canadian tertiary hospital.

**Methods:**

Single-day audits were undertaken on the pediatric ward (PW), pediatric emergency department (ED), and maternal services (MS). Participants (child or caregiver proxy) reported hospital pain experiences in the preceding 24 h; medical records were reviewed for assessment and treatment.

**Results:**

Among 84 participants, pain prevalence ranged from 75% to 88%; mean pain intensity ranged from 5.7 to 6.5/10. Prevalence of moderate to severe pain was 78% on PW, 65% in ED, and 55% on MS; needle pokes were the most frequent cause of worst pain. Documentation of pain assessment varied by setting (PW, 93%; ED, 13%; MS, 0%). Documented maximum pain scores were significantly lower compared to participant report (mean difference 4.5/10, SD 3.1, *P* < 0.0001). A total 29% (6/21) of infants with heel lance or injection received breastfeeding or sucrose, and 29% (7/24) of participants receiving other needle procedures had documented or reported topical lidocaine use. All participants on MS underwent needle procedures.

**Conclusions:**

Pain is experienced commonly by infants and children in PW, ED, and MS. Pain assessment documentation is not routine and underestimates participant report. Evidence-based pain management strategies are underutilized. An institution-wide quality improvement approach is required to address pain care. Pain assessment and needle pain prevention and treatment should be prioritized in these pediatric acute care and newborn care settings.

## RÉSUMÉ

**Contexte**: Les expériences douloureuses sont courantes chez les enfants hospitalisés. Les conséquences biopsychosociales négatives à long terme d'une douleur sous-traitée sont reconnues.

**Objectifs**: L'étude compare la prévalence, l'évaluation et le traitement de la douleur comme premières étapes pour améliorer les soins de la douleur dans un hôpital tertiaire canadien.

**Méthodes**: Des audits d'une journée ont été effectués dans les services de pédiatrie (SP), les services d’urgence pédiatrique (SU) et les services maternels (SM). Les participants (enfant ou soignant) ont rapporté des expériences de douleur à l'hôpital au cours des 24 heures précédentes; les dossiers médicaux ont été examinés pour évaluation et traitement.

**Résultats**: Parmi les 84 participants, la prévalence de la douleur variait de 75 % à 88 % ; l'intensité moyenne de la douleur variait de 5,7 à 6,5/10. La prévalence de la douleur modérée à sévère était de 78 % pour les services de pédiatrie, 65 % pour les services d’urgence pédiatrique et 55 % pour les services maternels ; les piqûres étaient la cause la plus fréquente des pires douleurs. La documentation de l'évaluation de la douleur variait selon le contexte (SP 93 % ; SU 13 % ; SM 0 %). Les scores maximaux de douleur documentés étaient significativement inférieurs à ceux rapportés par les participants (différence moyenne 4,5 / 10, SD 3,1, P < 0,0001). Au total, 29 % (6/21) des nourrissons ayant reçu une piqûre au talon ou une injection ont reçu un allaitement ou du saccharose, et 29 % (7/24) des participants recevant d'autres procédures d'injection avaient documenté ou signalé une utilisation topique de lidocaïne. Tous les participants des services maternels ont subi une procédure impliquant une aiguille.

**Conclusions**: La douleur est couramment ressentie par les nourrissons et les enfants dans les services de pédiatrie, les services d’urgence pédiatrique et les services maternels. La documentation relative à l’évaluation de la douleur n'est pas systématique et sous-estime la douleur rapportée par les participants. Les stratégies de prise en charge de la douleur fondées sur des données probantes sont sous-utilisées. Une approche d'amélioration de la qualité à l'échelle de l'établissement est nécessaire pour aborder les soins de la douleur. L'évaluation de la douleur et la prévention et le traitement de la douleur causée par les aiguilles devraient être prioritaires dans ces établissements de soins pédiatriques aigus et de soins aux nouveau-nés.

## Introduction

Inadequate treatment of pain in childhood has long-term negative biopsychosocial consequences.^1–8^ Children’s pain memories strongly influence future procedural pain experiences and may affect transition to chronic pain.^9^ For example, painful experiences are associated with reduced efficacy of analgesia for future procedures and increased morbidity and mortality.^4,5,7,8^ Because needle fear affects 10% to 25% of adults and is associated with avoidance of medical care, improved needle pain management should be prioritized in childhood.^5,10^ Despite published guidance on best practice for pain prevention, assessment, and treatment during pediatric hospitalization, pain prevalence remains high.^1,3,11–13^ Approximately 75% of pediatric patients experience pain while in hospital, and up to 30% experience severe pain.^14^ Painful diagnostic procedures and therapeutic interventions, including needle pokes, are common, as are painful medical or surgical conditions, such as trauma.^3^ A large Canadian study demonstrated that the majority of hospitalized children on pediatric units underwent a painful procedure in the preceding 24 h.^2^

Use of evidence-informed, developmentally appropriate pain assessment processes and multimodal pain treatment and prevention protocols is associated with improved outcomes.^1,15^ Effective pain management depends on regular pain assessment and follow-up to evaluate response to pain management interventions. Use of patient self-report measures is emphasized due to the subjective nature of pain experience and concerns about lack of regular pain assessment and documentation in clinical care.^16^ A multimodal approach to pain management combines psychological, pharmacological, and physical strategies to minimize pain and distress.^3^ For example, a multimodal approach to blood draw typically includes preparation and distraction combined with use of topical numbing cream (NC) and upright or comfort positioning. For infants, skin-to-skin contact, breastfeeding, or oral sucrose is recommended for heel lancing and other simple procedures.^3,14,17–20^ However, unless pain management is institutionalized as a core value, inconsistent practice limits success and sustainability.^1^

Five principles that support evidence-informed pain prevention, assessment, and treatment practices within health facilities, have been described by ChildKind International, a not-for-profit organization that emphasizes children’s pain care as a cornerstone of compassionate care.^1^ The key principles are institutional commitment; ongoing educational programs for staff, patients, and their caregivers; developmentally appropriate pain assessment processes; multimodal pain prevention and treatment protocols; and regular institutional self-monitoring.^1^ Together, these principles provide a framework to support sustained institutional change within a continuous quality improvement framework.

Several major children’s hospitals have undertaken benchmarking studies to assess both pain prevalence as an outcome and the processes that underpin it, such as pain assessment and pain treatment.^14,15,21,22^ As a first step to improving pain care within a quality improvement framework, the aim of this study is to benchmark pain prevalence, assessment, and treatments in a Canadian tertiary care hospital. Benchmarking current practice serves to raise awareness, identify gaps in care, and provide a baseline for follow-up evaluation.

## Methods

The University of Saskatchewan Research Ethics Board (Bio #2621) approved this study. Verbal consent to participate was obtained from a parent or legal guardian. With permission, this study was adapted from the Minneapolis Children’s Hospital;^14^ similar audits have been effective to benchmark and measure improvements in pediatric pain care.^14,15,21,22^ This study utilized a multimethod approach, with a brief in-person survey administered verbally to eligible children and caregivers followed by a chart audit. The survey and data extraction were conducted with Research Electronic Data Capture, a secure web-based software platform.^23,24^ The researcher notified a health care professional responsible for the child’s care if pain was being experienced at the time of the survey.

This tertiary hospital serves approximately 360,000 people in the surrounding area and is a provincial pediatric referral center for children from birth to age 16 years. Clinical nursing policy established in 2002 and updated in 2012 requires pain assessment with a developmentally appropriate scoring tool at time of admission or health care interaction, with vital signs, when the child is at risk for pain and within 1 h of a pain management intervention. Pain, intensity is measured using an 11-point numerical rating scale (where 0 = *no pain* and 10 = *worst pain possible*).^25^ If the child is nonverbal, Faces Pain Scale–Revised or the Face, Legs, Activity, Cry, Consolability scale is used.^26–27^ A multidimensional qualitative assessment and multimodal approach to pain management is described in the policy. Lidocaine cream (Maxilene 4, RGR Pharma, Windsor, ON) is available on formulary. A medical directive supports administration of nurse- or caregiver-administered 24% sucrose, and documentation is required. A standard newborn admission order set on maternal services (MS) includes sucrose for minor invasive procedures when breastfeeding is not possible.

### Recruitment

Children from birth to age 16 years with a caregiver/guardian present were eligible to participate. All children listed on the morning census from the pediatric ward (PW) and MS ward were eligible. Children presenting in the pediatric emergency department (ED) were identified throughout the day from the registration desks. Children who met eligibility criteria were invited to participate in the survey and chart audit. There were no exclusion criteria. Recruitment occurred during single-day (8-h shift) periods on PW, ED, and MS during June and July of 2018. During the study period, recruitment posters were posted in common areas visible to staff, patients, and families. Clinical staff were not informed in advance when audits would occur. To capture patients under the care of a variety of staff, recruitment occurred over 3 days for the PW, 4 days for the ED, and 2 days for MS. Three attempts were made to locate children and caregivers who were not at the bedside.

### Child/Caregiver Survey

The survey contained 18 questions, including demographic characteristics, length of stay and reason for visit, and whether or not pain had been experienced during the prior 24 h (or since time of admission if less than 24 h). If the patient had experienced pain, inquiry continued about cause of worst pain, pain intensity, usefulness of pain management strategies offered, and satisfaction with pain management. Children aged 5 to 16 years were interviewed directly if their parent or guardian believed they were capable of understanding and answering questions about pain. The researcher read out each survey question and selected the survey response that matched the participant’s answer using a tablet at the bedside. For the purpose of this study, needle poke was defined as any procedure that broke the skin (e.g., venipuncture, heel lancing, intravenous start, port access, lumbar puncture, injection). History of chronic pain was determined by asking, “Before this admission, did you/your child usually have pain or anything that hurt routinely?”

#### Pain Measures

Pain intensity was evaluated by caregiver proxy report or child self-report with a numeric rating scale scored on a scale from 0 (*least pain possible*) to 10 (*worst pain imaginable*).^14^ Children too young to complete the numeric scale were offered the Faces Pain Scale–Revised.^26^

#### Satisfaction with Pain Management

Child/caregiver satisfaction with treatment of pain was measured using an 11-point Likert scale ranging from 0 (*not satisfied*) to 10 (*very satisfied*). Participants were asked, “How satisfied are you overall with how your/your child’s pain was treated?”

### Chart Audit

A data collection template was utilized to extract data on pain assessment and management from participants’ medical records. Clinical data sheets, nursing notes, medication reconciliation records, and practitioner notes were reviewed to identify pain assessment and treatment. Information included diagnoses; pain scores; name, route, and frequency of pain medication ordered/administered; and anesthesiology and interprofessional team consultation (e.g., health psychology, recreational therapy). Medications were categorized as follows: simple analgesia (e.g., acetaminophen, ibuprofen, ketorolac, and naproxen), opioid (e.g., morphine, fentanyl, and hydromorphone), adjuvant (e.g., gabapentin, low-dose ketamine, midazolam, diazepam, lorazepam), and topical anesthetic. Chart documentation was assessed on all participants, whether or not they reported pain.

### Analyses

Analyses were stratified and presented by location to assess diversity. Descriptive statistics (e.g., frequencies and percentages) were calculated to describe characteristics of the children; the occurrence of pain; the prevalence of maximum pain rated as none, mild (1–3/10), moderate (4–6/10), and severe (≥7); pain strategies utilized; and charted pain documentation. Proportions of specific strategy use among participants undergoing needle poke procedures within the past 24 h who were eligible for NC (e.g., venipuncture, intravenous start, port access, lumbar puncture), breastfeeding/sucrose (i.e., heel lancing, routine newborn injections), or infant-specific comfort strategies (e.g., swaddling, rocking) were calculated overall. The use of these strategies was also reassessed among those indicating that needle pain was the cause of their worst pain.

In comparative analysis, means and standard deviations were calculated for both maximum pain scores (both reported and charted) and satisfaction with pain management; means were also calculated for helpfulness of pain management strategies. Comparisons of mean scores across units were undertaken using analysis of variance; pain frequencies, proportion with documentation, and proportions experiencing needle procedures were compared between the units using chi-square testing. Select subgroup comparisons of pain scores were made using the *t*-test for means and Fisher’s exact test for proportions. Maximum pain scores, as reported by both child/caregiver and chart, were compared using the paired *t*-test; similarly, the proportions of children experiencing moderate to severe pain according to child/caregiver report and chart documentation were compared using McNemar’s exact test. For all above comparisons of mean pain scores, nonparametric testing equivalents (Kruskal-Wallis, Mann-Whitney *U*, and Wilcoxon signed-rank testing) were undertaken to support conclusions based on initial parametric methods that assume normality. Results were considered significant at the 95% confidence level. IBM SPSS Statistics for Windows, v27 was used for all analyses.^28^

## Results

### Participant Sample

**Figure 1.**
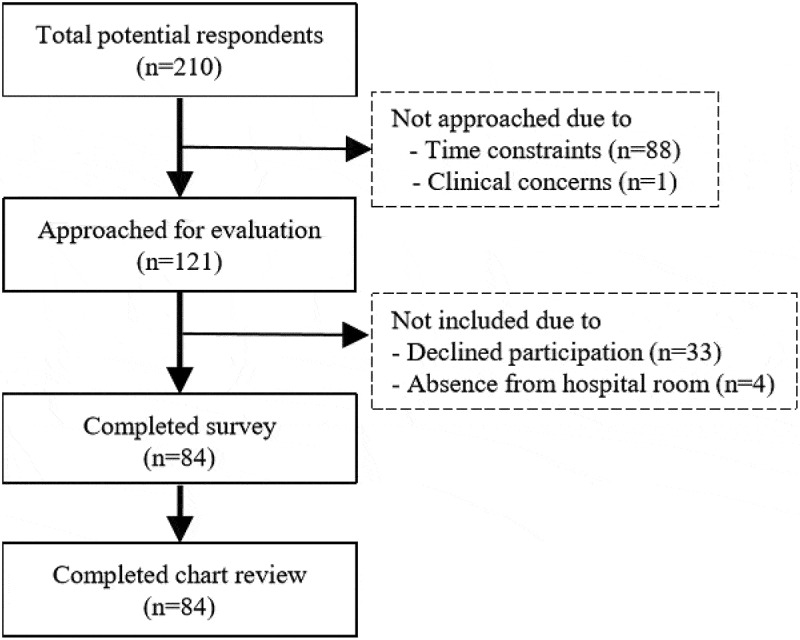
Study sample

See Figure 1 for a summary of the participant sample. Among respondents approached, 84/121 (69%) participated.

### Participant Characteristics

In total, 84 children from birth to 16 years of age were included in the study sample. Child demographic characteristics, respondent demographic characteristics, length of stay, reason for the visit, and history of chronic pain are presented in Table 1.

**Table 1.** Sample characteristics (*N* = 84)

	Ward (*n* = 41)	Emergency (*n* = 23)	Maternal services (*n* = 20)
Time in hospital at survey ≥24 h, *n* (%)	34 (83)	0	14 (70)
Respondent, *n* (%)			
Child	14 (34)	12 (52)	0
Parent/caregiver^a^	27 (66)	11 (48)	20 (100)
Language, *n* (%)			
English	32 (78)	17 (74)	15 (75)
Other	9 (22)	6 (26)	5 (25)
Patient sex, female, *n* (%)	23 (56)	12 (52)	6 (30)
Patient age, years, *n* (%)			
<1	8 (20)	2 (9)	20 (100)
1 to <5	10 (24)	9 (39)	0
5 to 16	23 (56)	12 (52)	0
Patient, history of chronic pain, *n* (%)	15 (39)^b^	1 (5)^c^	NA
Patient, reason for hospital visit, *n* (%)			
Acute illness/infection	17 (42)	15 (65)	0
Known disease/prematurity	10 (24)	0	0
Surgery/trauma	8 (20)	7 (30)	0
Other	6 (15)	1 (4)	20^d^ (100)

^a^Most parent/caregiver respondents were mothers; father/other counts were less than five in all three groups.

^b^Chronic pain status not available in three patients.

^c^One patient missing chronic pain status and not applicable to one child less than 1 year of age.

^d^All patients born on the maternal service.

NA = not applicable because child(ren) less than 1 year of age.

### Pain Prevalence and Assessment

**Figure 2.**
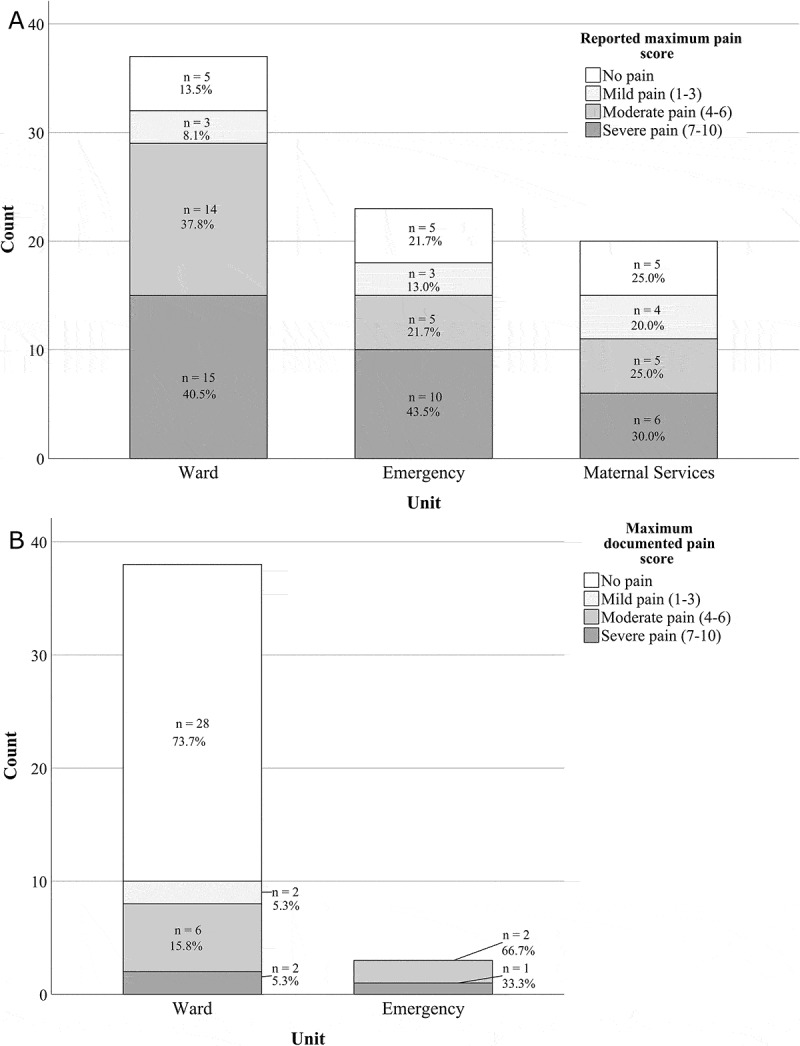
(A) Reported maximum pain score frequencies by category. Pain scores were not reported by four pediatric ward patients. (B) Maximum documented pain score by category. Three children on the ward, 20 children in the pediatric emergency department, and all 20 children on maternal services did not have pain score documentation

Eighty-two percent (69/84) of participants reported experiencing pain in the past 24 h or since arriving at hospital if less than 24 h. We present the distribution of participants by clinically relevant pain category in Figure 2. Frequencies of reported maximum pain score and maximum documented pain score are presented for each clinical unit.

A more detailed description of pain experiences is presented in Table 2. Pain prevalence and mean pain intensity during the previous 24 h were similar across settings (PW, 88%; ED, 78%; MS, 75%; *P* = 0.40, and PW, 6.3; ED, 6.5; MS, 5.7; *P* = 0.65). Among those reporting pain, minimum scores were 2, 2, and 1 across the units respectively; all units had maximum scores of 10.

**Table 2.** Pain prevalence, intensity, cause of worst pain, and documentation during the previous 24 h or since admission if <24 h

	Ward (*n* = 41)		Emergency (*n* = 23)		Maternal services (*n* = 20)	
	*n* (%) Pain intensity score, mean (SD)		*n* (%) Pain intensity score, mean (SD)		*n* (%) Pain intensity score, mean (SD)	
Patient-reported pain metrics, among patients with pain						
Worst pain reported, all causes^a^	36 (88)	6.3 (2.2)	18 (78)	6.5 (2.5)	15 (75)	5.7 (2.8)
By specific cause^b^						
Needle pokes	10 (28)	7.5 (2.1)	3 (17)	8.3 (1.5)	14 (93)	5.4 (2.7)
Surgery	4 (11)	NR^c^	0		0	
Procedure	4 (11)	6.5 (1.7)	3 (17)	4.0 (2.6)	1 (7)	NR
Acute illness/infection	6 (17)	5.2 (1.3)	8 (44)	7.5 (1.9)	0	
Treatment for known disease	5 (14)	4.4 (2.5)	0		0	
Trauma/injury/other medical	7 (19)	6.7 (2.3)	4 (22)	5.0 (2.0)	0	
Chart-reported pain metrics, all patients						
Maximum pain intensity documented^d^	38 (93)	1.4 (2.6)	3 (13)	6.0 (1.0)	0	

^a^Bracketed percentages indicate proportion of group reporting pain. Five in each group did not have pain.

^b^Bracketed percentages indicate proportions of those with pain who indicated the specific modality as the cause of their greatest pain.

^c^Although surgery was responsible for the most pain in four participants, worst pain score was only available for one.

^d^Bracketed percentages indicate proportion of group with documented pain score.

NR = not released for privacy.

Moderate to severe pain was reported by 78% (29/37), 65% (15/23), and 55% (11/20) of all patients on PW, ED, and MS, respectively (*P* = 0.18); four patients on PW indicating pain did not provide a pain score. Among those patients with pain, frequencies for causes of worst pain, again with percentages and average maximal intensity scores, are also shown in Table 2. Overall, moderate to severe pain was most commonly due to needle pokes (23/55, 42%).

In additional subgroup analysis, mean patient-reported intensities did not differ between those who did and did not consider English as their first language (6.1 [SD 2.4] versus 6.6 [SD 2.6], *P* = 0.46). Corresponding proportions for moderate to severe pain were 84% and 86% (*P* > 0.99), respectively. Maximal pain scores also did not differ between those with surgery/trauma as their reason for admission and those admitted for other reasons (6.2 [SD 2.6] versus 6.2 [SD 2.4], *P* = 0.98). Moderate to severe pain was reported by 77% and 87% (*P* = 0.41), respectively.

The presence of pain score documentation in the chart varied markedly between units (*P* < 0.0001) and is presented in Table 2. On the PW, there were sufficient data from chart audit to compare maximal documented pain scores to patient reported scores for 30 participants who reported experiencing pain during the study period. Maximum scores differed between child/caregiver report and chart documentation by an average of 4.5/10 (SD = 3.1, *P* < 0.0001), with higher scores reported by children/caregivers. Among the 27 participants on the PW with charted scores who reported moderate to severe pain, this difference was 4.8/10 (SD 3.1, *P* < 0.0001), again with higher pain scores reported by children/caregivers. Participants who reported moderate to severe pain constituted 90% (27/30) of this group; however, chart reporting only documented 7 children with moderate to severe pain among the 30 (23%, *P* < 0.0001). Two participants on the PW reporting moderate to severe pain had no charted pain scores.

### Pain Management Strategies

Frequency and helpfulness of pain management strategies used by children were rated by survey participants and are reported in Table 3.

**Table 3.** Strategies used by children with pain during the previous 24 h or since admission if <24 h and their helpfulness as reported by patients/caregivers

	Ward^a,b^ (*n* = 41)		Emergency^b^ (*n* = 23)		Maternal services^b^ (*n* = 20)	
	*n* (%) Pain intensity score, mean (SD)		*n* (%) Pain intensity score, mean (SD)		*n* (%) Pain intensity score, mean (SD)	
	Use, *n* (%)	Helpfulness, mean (SD)	Use, *n* (%)	Helpfulness, mean (SD)	Use,*n* (%)	Helpfulness, mean (SD)
Child participation	15 (42)	7.9 (2.7)	3 (17)	6.3 (2.1)	0	
Caregiver participation	30 (83)	8.1 (2.1)	14 (78)	7.2 (2.9)	13 (87)	8.3 (2.0)
Distraction	24 (67)	5.3 (3.5)	9 (50)	4.2 (2.9)	6 (40)	7.7 (3.8)
Positioning	19 (53)	6.9 (2.6)	13 (72)	6.5 (2.8)	11 (73)	7.8 (1.9)
Pain medicine	21 (58)	8.4 (2.1)	13 (72)	7.1 (2.7)	1 (7)	NR
Topical numbing cream^c^	7 (19)	7.9 (2.3)	1 (6)	NR	1 (7)	NR
Education/information/preparation	20 (56)	6.7 (3.7)	11 (61)	5.1 (3.4)	0	
Warm/cold pack	10 (28)	7.4 (2.7)	2 (11)	7.5 (2.1)	5 (33)	7.8 (1.9)
Epidural/blocks	0		0		0	
Other	1 (3)	NR	3 (17)	9.3 (1.2)	4 (27)	7.8 (3.3)
Infant-specific strategies^d^						
Infant comfort (swaddling, rocking, etc.)	5 (83)	8.2 (2.5)	0		8 (53)	9.3 (1.2)
Breastfeeding/sucrose	4 (67)	9.3 (1.5)	0		5 (33)	8.6 (1.7)
Pacifier	4 (67)	7.8 (4.6)	0		0	

^a^One ward participant reported receiving no pain relief strategies.

^b^Patients may have received multiple strategies; integrative medicine strategy results are not presented because of small cell sizes.

^c^Among participants reporting topical numbing cream use, nature/timing of related procedure was unclear for two respondents.

^d^Only evaluated in 22 infants within 12 months of age who had pain as per parental report (6 on ward, 1 in emergency, and 15 on maternal services); patients may have received multiple strategies.

NR = not released for privacy.

Scheduled and unscheduled pharmacologic pain management interventions ordered for all children are reported in Table 4. No children on MS had pharmacologic analgesia ordered. Six children (6/36, 17%) on the ward and five children in ED (5/18, 28%) who reported pain had no pharmacologic analgesia ordered; among these children with no orders, four in each group reported moderate to severe pain (PW 4/6, 67%; ED 4/5, 80%). Most of the children in PW and ED with pain who had no analgesia ordered identified a cause other than needle procedure as the source of their worst pain (four in each group).

**Table 4.** Scheduled and unscheduled pharmacologic pain management interventions ordered for all children, those who experienced any pain, and those who experienced moderate to severe pain in the pediatric ward or emergency department during the past 24 h or since admission if <24 h

	All children^a^		Children who reported pain		Children who reported pain ≥4	
	Ward	Emergency	Ward	Emergency	Ward	Emergency
	*n* = 41	*n* = 23	*n* = 36	*n* = 18	*n* = 29	*n* = 15
Acetaminophen, *n* (%)	25 (61)	7 (30)	23 (64)	5 (28)	19 (66)	4 (27)
NSAIDs, *n* (%)	14 (34)	10 (43)	12 (33)	8 (44)	11 (38)	7 (47)
Opioids, *n* (%)	12 (29)	4 (17)	11 (31)	4 (22)	9 (31)	4 (27)
Adjuvant analgesia, *n* (%)	5 (12)	3 (13)	4 (11)	3 (17)	3 (10)	3 (20)
Topical anesthetic prior to needle pokes	9 (22)	0	9 (25)	0	9 (31)	0

^a^No children on maternal services had pharmacologic analgesia ordered. Total number of patients receiving the individual agents within each column may sum to more than the total for the category because some patients received more than one agent with each class.

NSAID = nonsteroidal anti-inflammatory drug.

Forty-five participants reported a needle procedure in the previous 24 h (PW, 16/41, 39%; ED, 9/23, 39%; MS, 20/20, 100%; *P* = 0.0001). Twenty-one received a heel lance or newborn injection (e.g., vitamin K injection), with 19 of these taking place on MS. Twenty-four received other skin-breaking procedures (e.g., blood draw, intravenous start, port access). Of those receiving heel stick or newborn injections, six (6/21, 29%) reported breastfeeding or sucrose and ten (10/21, 48%) reported infant comfort strategies. Of those with a heel stick procedure as the cause of worst pain, 40% (6/15) had breastfeeding or sucrose provided and nine (9/15, 60%) received infant comfort. Of those who were eligible for NC, six (6/24, 25%) had orders and seven (7/24, 29%) had application documented in the chart or use reported; two other participants indicated NC use (Table 3) but circumstances related to use were unclear. Among those with needle pain as worst pain who were eligible for NC, five (5/12, 42%) had lidocaine ordered and six (6/12, 50%) had lidocaine application documented in the chart or use reported.

There were no significant differences in the average self-reported pain management satisfaction between the three wards (PW, 7.9/10; ED, 7.9/10; MS, 8.5/10; *P* = 0.59).

## Discussion

Of 121 patients registered in three pediatric care settings (PW, ED, MS), 84 children from birth to 16 years of age and their parents completed the survey. Pain prevalence during the previous 24 h ranged from 75% to 88%, and mean pain intensity was similar across settings. Overall, needle pokes were the most common cause of moderate to severe pain. Proportion with at least one documented pain score ranged from 0% on MS and 13% on ED to 93% on PW. Documented maximum pain scores were significantly lower compared to participant report. Overall, more than half of participants experienced a needle procedure during the study period. NC was not ordered consistently, and in some cases, use was reported without orders or documentation. Overall, infant-specific strategies were underutilized. Newborns on MS were significantly more likely to experience a needle procedure compared to participants on other units.

### Pain Prevalence and Assessment

The prevalence of pain and its typical causes, intensity, and underreporting seen in our sample are not surprising when compared to the findings of similar studies.^2,14,21,29^ In another tertiary Canadian institution, 77% of children experienced pain during admission and 64% had moderate or severe pain in the previous 24 h; only 27% had a pain score documented during that time.^21^ In a South African study, 87% of children admitted to an inpatient pediatric unit experienced pain and only 16% had a pain score documented during that time.^29^ Previous studies did not include maternal birthing care units where newborns receive routine care.^2,14,21,29^

Needle-related procedures are a frequent cause of worst pain among hospitalized pediatric patients.^2,29^ In our sample, needle pain was reported as the source of worst pain experienced by 39% (27/69) of children who experienced pain. Similarly, among those experiencing moderate to severe pain overall, the most common cause was needle pokes. In previous studies, hospitalized children most frequently reported that worst pain was caused by needle pokes with prevalence of 34% to 40%, followed by surgery or trauma/injury.^14,29^ This proportion was observed to vary between the clinical units in our study, lower in the ED where worst pain from acute illness/infection was more common and higher on the MS where needle pain accounted for nearly all worst pain experiences.

Underestimation of pain by health professionals has been recognized in multiple pediatric studies.^14,21,30–34^ In our sample, pain assessment was not documented on MS, and documentation in ED was limited. In similar studies on inpatient pediatric units, documentation rates for pain assessments and treatments during a 24-h audit ranged from 58% to 78%.^2,14^ We found documentation to be highest on PW, where pain scores are obtained with routine vital signs every 4 h; however, maximum scores between child/caregiver and chart significantly differed. Although correct use of validated pain measures is expected in clinical settings, health care professionals may have relied on their own overall impression of the child’s pain rather than involving the child/caregiver in pain assessment and using validated scales.^34–36^ It is also possible that pain assessment may not have corresponded with pain triggers (e.g., blood draw, intravenous start, therapies, end of dose effect).

### Pain Treatment

Overall, fewer than half of infants undergoing skin-breaking procedures received breastfeeding or sucrose, although use of infant comfort strategies was more common. On MS, where newborns receive needle procedures routinely for universal screening, management of needle pain on the first procedure is possible. Parental education has been demonstrated to reduce children’s procedure-related distress and fear, and parental presence is associated with increased use of pain management strategies and reduced distress and fear.^19^ Alongside universal screening, the opportunity arises to educate parents and caregivers on effective strategies and to model appropriate techniques that they may implement in the future (e.g., at immunization).^3,19^

NC use has been found to be low prior to implementation of institution-wide protocols in other studies.^21,30,37^ In our study, only one quarter of infants and children eligible for NC (i.e., had a needle procedure) had it ordered. Some participants reported use but there was no corresponding practitioner order or record of administration, highlighting the importance of gathering audit data directly from patients and families. Arguably, all children in acute care settings should have NC ordered (if no contraindication), in case the need for blood draw or injection arises.^3^ For optimal pain management, a plan to address pain and anxiety before initiation of any procedure is advised.^6^

Of children reporting pain in the ED, nonsteroidal anti-inflammatory drugs were most commonly prescribed, followed by acetaminophen and opioids. Pain assessment was documented less commonly than on PW. Moderate to severe pain was common among the participants who had no analgesia prescribed. Considering that in ED, worst pain was more frequently attributed to acute illness or infection followed by trauma, injury, and other medical causes, a different approach may be required. Introduction of a stepwise approach to pharmacologic pain management with a pain treatment protocol may be particularly helpful in this setting.^38^ Although needle pain was not at the forefront during the ED admission, the importance of needle pain prevention on the first exposure cannot be overstated because this sets the tone for future needle pain experience in hospital.^4,5,7,8^

Despite the high pain prevalence and prevalence of moderate to severe pain, we observed that most participants were satisfied with their pain management. Similarly, in other pediatric tertiary hospitals, participants were typically satisfied with pain management, despite high prevalence of moderate to severe pain.^14,30^ Shorter times to analgesia administration and perception that hospital staff listened to patients and caregivers may be factors associated with satisfaction.^14,39,40^

### Future Directions

We identified inconsistent pain assessment and treatment in pediatric acute care and newborn care settings in this hospital. However, institutional follow-up studies suggest that improvements are possible.^15,22,37,41^ An institution-wide commitment (policy) is required to support consistent and sustained practice across all departments.^1,22^ Considering the link between pain assessment and pain outcomes,^4,5,7,8^ as well as the high frequency of painful needle procedures observed in the study, we identify the need for improved pain assessment and needle pain management as two priorities for evidence-based protocol development. At the unit level, developmentally appropriate pain assessment and context-specific treatment protocols are required, along with simplified processes (including reminders) and follow-up audits that assess related unit-specific outcomes.^1,22^ Engaging both caregivers and staff, tailoring knowledge translation strategies to unit context, supporting unit leadership, and dedicating resources will be essential to achieve institutional change.^1,14,41–44^

### Limitations

Because our study investigated three clinical settings within our tertiary care center, results are not generalizable to intensive care or community-based hospital settings. Although this survey aimed to capture “usual” days at our institution, these findings may not represent the typical experience. Although staff were not informed on which days audits would occur, recruitment materials and questions from patients and families about the project may have affected staff assessment, treatment, or documentation practices. Selection bias may be present, with participants having characteristics, including pain experiences, that differ from those of nonparticipants. Pain reporting was indirect for several cases because a parent/caregiver provided proxy responses for 69% of children. Given that the survey was verbally administered, participants may not have been comfortable expressing dissatisfaction with pain management. The chart audit has the usual limitations of retrospective review; for instance, staff may have assessed pain and not documented it. It is also possible that curtailing the interview among patients who reported no pain underestimated effective pain management in place.

## Conclusions

This study benchmarks pain prevalence, assessment, and treatment as a first step to improve pain care in a Canadian tertiary care hospital. Moderate to severe pain affects half to three quarters of infants and children across all three units. Documentation of pain assessment is not routine in all settings; when documented, maximum pain scores are significantly lower compared to participant experience. Needle pokes are the most frequent cause of worst pain, and all newborns underwent needle procedures. The majority of infants and children undergoing skin-breaking procedures do not receive optimal pain care because simple, safe, evidence-based pain management strategies are underutilized. Pain assessment and needle pain management are top priorities to be addressed in these pediatric acute care and newborn care settings. An institution-wide quality improvement approach will be required.

## Data Availability

Due to the nature of this research, participants in this study did not agree for their data to be shared publicly, so supporting data are not available.
